# Morphology Evolution and Rheological Behaviors of PP/SR Thermoplastic Vulcanizate

**DOI:** 10.3390/polym11010175

**Published:** 2019-01-19

**Authors:** Qiang Wu, Jiafeng Fang, Minghuan Zheng, Yan Luo, Xu Wang, Lixin Xu, Chunhui Zhang

**Affiliations:** 1 College of Materials Science and Engineering, Zhejiang University of Technology, Hangzhou 310014, China; luoyan@zjut.edu.cn (Y.L.); gcsxlx@zjut.edu.cn (L.X.); 2 College of Engineering, Zhejiang A&F University, Hangzhou, 311300, China; 18857292977@163.com (J.F.); zmhzafu@163.com (M.Z.); 3 Zhejiang Liniz Advanced Materials Co. Ltd, Hangzhou, 311305, China; soglad@163.com (C.Z.)

**Keywords:** silicone rubber, polypropylene, thermalplastic vulcanizates, viscoelasticity, creep

## Abstract

The thermoplastic vulcanizates (TPVs) of polypropylene (PP)/silicone rubber (SR) were prepared by dynamic vulcanization (DV) technology. The mixing torque, morphology, viscoelasticity, and creep response of PP/SR TPVs were investigated by torque rheometer, scanning electron microscope (SEM), transmission electron microscope (TEM), rotational rheometer, and dynamic mechanical analysis (DMA). A mixing-torque study showed that torque change and dynamic-vulcanization time increased with SR content increasing in the DV process, but DV rate was independent of SR content. TEM images indicated that the phase inversion of PP/SR-60 TPV from bicontinuous to a sea–island structure took place in the DV process, and a hot press would break the rubber aggregates and shrink a large SR phase. Dynamic-strain measurement demonstrated that PP/SR TPVs exhibit a distinct “Payne effect”, which can be attributed to the destruction and reconstruction of SR physical networks. Complex viscosity indicated that SR content did not affect the processability of PP/SR TPVs at high shear rates. Furthermore, the creep deformation and recovery of PP/SR TPVs at solid and melt states were studied, respectively.

## 1. Introduction

Thermoplastic elastomers (TPEs) are polymers that exhibit rubbery properties at using temperature and melt processability above melt temperature. In general, there are two main kinds of TPEs, the block copolymer that has “soft” and “hard” segments, and the thermoplastic vulcanizate (TPV), prepared by dynamic vulcanization, where the curing of rubber occurs during mixing with plastic under a high temperature and shear [1]. Compared with block copolymers, TPV is more suitable for industrial applications. Dynamic-vulcanization (DV) technology was first reported by Gessler and Haslett [2] in 1962, and then further developed by Fischer [3] on polypropylene (PP)/ethylene propylene diene rubber (EPDM) TPVs. PP/EPDM TPVs were finally industrialized by Monsanto in 1981, and are the most studied TPV [4,5,6,7,8,9,10]. To achieve high or functional performance, various TPVs were prepared by mixing different plastic phases and rubber phases, such as polyamide 6 (PA6)/EPDM [11,12,13], PA12/bromobutyl rubber (BIIR) [14], PP/ethylene octene copolymer (EOC) [15,16], poly(lactide) (PLA)/natural rubber (NR) [17], PLA/ethylene-*co*-vinyl acetate (EVA) [18], PLA/bio-based polyester [19], and thermoplastic polyurethane (TPU)/ NR [20].

Silicone rubber (SR) is an elastomer composed of a Si–O–Si main chain, and widely used in the medical, electronics, construction, automotive, and food fields. Nowadays, due to SR’s low-temperature flexibility, high-temperature stability, and good biocompatibility, it was selected as a rubber phase to prepare TPVs such as PA/SR [21] and poly vinylidene fluoride (PVDF)/SR TPVs [22,23]. However, almost all SR-containing TPVs were prepared by using a peroxide curing system, which would leave byproducts and become an issue in food contact and medical applications. Besides the peroxide curing system, SR can be crosslinked by a platinum-based curing system, which is also called a hydrosilylation mechanism. The hydrosilylation mechanism refers to the additional reaction of Si–H bonds to carbon double bonds borne by poly(dimethylsiloxane) chains in the presence of a platinum catalyst, which has no byproducts and fast curing speed. In addition, compared to PA and PVDF, investigated in previous studies [21,22,23], PP is a semi-crystalline thermoplastic with good heat, oil, and chemical resistance, outstanding tensile properties, good processability, and low density and cost. Therefore, SR with a platinum-based cure system and PP were chosen to prepare PP/SR TPV by using dynamic-vulcanization technology. 

Compared with traditional polymer blends, the preparation of TPVs is more complex due to the simultaneous mixing of various compositions, and the crosslinking and breakup of the rubber phase. The composition ratio, the viscosity ratio of rubber and plastic, and the curing agents (curing rate and extent) could influence the DV process and determine the final morphology and properties of the TPV products. In order to control the final morphology, different feeding procedures [5,24,25] and electron-induced reactive processing [26] were studied. The preparation and microstructure property relationships have been well-reviewed by Ning et al. [1].

Therefore, in order to control the final structure and properties of PP/SR TPVs, morphology evolution and rheological behavior were investigated in this study. The specific objectives included studying the mixing torque and morphology evolution of PP/SR blends at different dynamic-vulcanization stages, and investigating the influence of the PP/SR ratio on the viscoelastic and creep behaviors of PP/SR TPVs.

## 2. Materials and Methods

### 2.1. Materials

The polydimethylvinyl siloxane (PMVS, *M*_w_ = 6×10^5^ g/mol), polymethylhydrosiloxane (PMHS, η_25°C_ = 80 mPa·s), Pt catalyst, and alkynol inhibitor were provided by Zhejiang Liniz Advanced Materials Co. Ltd., Hangzhou, China. PP (BA415E, *M*_w_=5.7×10^5^ g/mol, *M*_w_/*M*_n_ = 7.0) was purchased from Borealis, Vienna, Austria; its melt temperature was 164 °C, crystalline content was 58%, and flow index was 0.5 g/10 min (230 °C, 2.16 kg). The antioxidant (Irganox 1010) was supplied by Ciba Specialty Chemicals Inc, Basel, Switzerland.

### 2.2. Preparation of PP/SR TPVs

SR was crosslinked by the hydrosilylation mechanism, and the curing reaction of SR can be found in our previous study [27]. PP/SR TPVs with a weight ratio of 70/30, 60/40, 50/50, 40/60 and 30/70 were prepared by using HAAKE PolyLab QC (Karlsruhe, Germany), and were named as PP/SR-30, PP/SR-40, PP/SR-50, PP/SR-60, and PP/SR-70, respectively. All the mixing parameters were carried out at 180 °C and 80 rpm rotor speed. At first, PP, PMVS, PMHS, alkynol inhibitor, and antioxidant were added and mixed in the chamber. Then, after mix torque reached a stable value, which indicated that the blends were mixed to uniformity, the Pt catalyst was added into the chamber to prepare dynamically crosslinked blends. Until the final stable torque was reached, the blends continued to mix for 3 min. Subsequently, the PP/SR blends were removed. The formulations of PP/SR TPVs are shown in Table 1. The constant volume method of HAAKE was used to prepare the PP/SR TPV samples. Because the chamber volume was 69 cm^3^, the filling level was 80%, and the density of PP and SR were very close (0.9 and 1.0 g/ cm^3^, respectively), the total weight of each PP/SR TPV formulation was 55 g.

All the specimens for mechanical, rheological, and DMA testing were compression-molded by using a hydraulic press (Daylight Press, Huzhou Dongfang Machinery Company, Huzhou, China). First, the PP/SR TPVs were placed into a square mold (200 mm × 200 mm) with 2 mm thickness and preheated for 3 min to melt the PP at 180 °C; then, the samples were hot-pressed under a pressure of 10 MPa for 8 min, and pressure was released 3 times to remove the bubbles. After the hot press, the samples were placed in another press at room temperature to form. Finally, the PP/SR TPV rectangular sheet was cut into a dumbbell and circular specimens by standard mold knives. The dumbbell specimen was type 4 in ISO 37: 2017 for the mechanical test, and the circular specimens were 25 mm in diameter for the rheological test. DMA samples were a rectangular strip with 80 mm × 10 mm × 2 mm (length × width × thickness).

### 2.3. Characterizations

#### 2.3.1. Morphology Characterization

A TM3030 (Hitachi, Tokyo, Japan) scanning electron microscopy (SEM) was used to study the surface topography of the cryogenic fractured PP/SR TPV specimens. Before SEM observation, the fracture surface was etched by cyclohexane at 100 °C for 10 min and ultrasonic-treated to remove the rubber particles. Afterward, the etched surface was coated with gold and subjected to SEM observation. 

Transmission electron microscopy (TEM) images of the PP/SR blends were obtained with a JEM-1200EX. A small amount of PP/SR blends (less than 0.2 g) at different mixing stages were taken out of the HAAKE chamber and rapidly quenched using liquid nitrogen to fix the phase structure [28]. Then, the samples were sliced into thin films with a thickness of about 100 nm by using a cryo-ultramicrotome (Leica EM UC7; Germany) at −150 °C.

#### 2.3.2. Dynamic Rheological Measurements 

A strain-controlled rheometer (ARES-G2, TA Instruments, New Castle, Delaware, USA), equipped with a plate-serrated fixture 25 mm in diameter, was used to study the dynamic rheological behavior of PP/SR TPVs. Dynamic strain sweep tests were conducted at a frequency of 10 rad/s with varying strain, from 0.01% to 100% at 200 °C to find the linear viscoelastic region. The frequency sweep tests were performed from 0.025 to 100 rad/s at strain amplitude within the linear viscoelastic region of 1% at 200 °C. 

#### 2.3.3. Creep Measurement

Creep and its recovery tests were measured by dynamic mechanical analysis (DMA800, TA Instruments, New Castle, Delaware, USA) for PP/SR TPVs in solid state at 40 °C, using ARES-G2 for PP/SR TPVs in melt state at 180 °C. For DMA measurements, PP/SR TPV solid samples were subjected to a constant stress of 1 MPa for 20 min, then recovered for 10 min by using DMA tension mode at 40 °C. For ARES-G2 measurement, PP/SR TPV melt samples were subjected to a constant stress of 100 Pa for 200 s, then recovered for 200 s by using shear mode at 180 °C.

## 3. Results and Discussions

### 3.1. Mixing Torque of PP/SR TPVs

**Figure 1:**
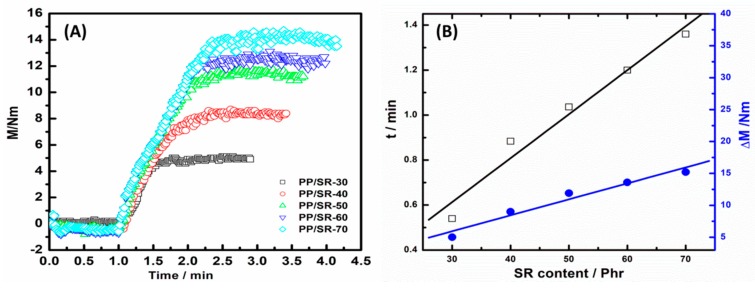
(**A**) Mixing torque change of polypropylene (PP)/silicone rubber (SR) blends in dynamic vulcanization process and (**B**) the relationship between SR content and torque change (Δ*M*) and dynamic vulcanization time (*t*).

Figure 1A presents the torque change after the Pt catalyst was added into the chamber. To better describe the torque change of the SR crosslink, the premixing balance torques of PP, PMVS, PMHS, and the antioxidant were normalized to 0, and the premix processes are not shown here. It can be found that the mixing torque immediately increased and then reached a final stable value after the Pt catalyst was added into the chamber, which indicated the formation of SR phase crosslinking [1]. All the dynamic-vulcanization behaviors of PP/SR blends showed similar torque changes, and the final torque and dynamic-vulcanization time increased with the increase of SR content. Moreover, during the dynamic-vulcanization process, the slopes of torque and time were similar, that is, 10.4, 10.2, 11.1, 10.9 and 11.8 N·m/min for PP/SR-30, PP/SR-40, PP/SR-50, PP/SR-60, and PP/SR-70, respectively. Figure 1B shows a good linear relationship between SR content and torque change or dynamic-vulcanization time. Torque change and slopes corresponded to the vulcanization degree and dynamic-vulcanization rate of the SR phase; hence, it indicated that vulcanization degree and dynamic-vulcanization rate were independent of SR content. This phenomenon also demonstrated that PP did not influence the SR crosslink reaction.

### 3.2. Morphology of PP/SR TPVs

**Figure 2:**
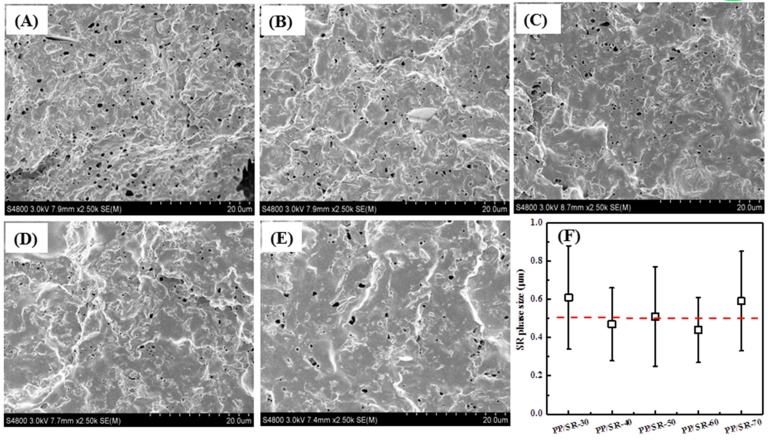
SEM images of etched surfaces for different polypropylene (PP)/silicone rubber (SR) thermoplastic vulcanizates (TPVs). (**A**) PP/SR-30, (**B**) PP/SR-40, (**C**) PP/SR-50, (**D**) PP/SR-60, (**E**) PP/SR-70 and (**F**) SR phase size statistical result.

Figure 2 shows the SEM images of the etched surfaces of PP/SR TPVs and the SR-phase size statistical result. It can be seen in all SEM images that many small holes appeared, represented as the SR phase, due to the SR phase being etched and removed by cyclohexane. The size statistical result found that SR size in the PP/SR TPV was about 0.5 μm, and was independent of SR content, which may be attributed to the dynamic-vulcanization rate being independent of SR content and the process condition being the same for all PP/SR TPV preparations. Furthermore, Chatterjee et al. [21] and Wang et al. [22] studied PVDF/SR and PA/SR TPVs, and showed similar SEM images of the TPV surface, with an SR size from 0.5 to 8 μm and about 2 μm, respectively. The SR size in our study was much lower than those in their studies, which may be attributed to PP and SR having better compatibility than PVDF/ SR and PA/ SR blends.

**Figure 3:**
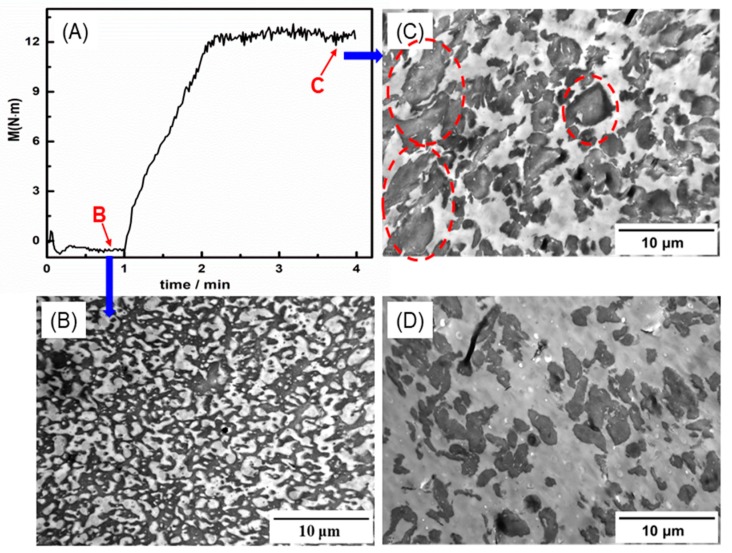
The relationship between different stages of polypropylene (PP)/silicone rubber (SR)-60 preparation and its morphology evolution, (**A**) Mixing torque change of PP/SR-60 preparation, (**B**) TEM image of PP/SR-60 blend at point B, (**C**) TEM image of PP/SR-60 thermoplastic vulcanizate at point C and (**D**) TEM image of PP/SR-60 TPV after hot press.

At different stages of dynamic-vulcanization processing, PP/SR blends exhibited different phase morphologies. Therefore, the PP/SR-60 blend was chosen to study the morphology evolution in processing. According to the torque-change plot of PP/SR-60 prepare process shown in Figure 3A, specimens at two positions (B and C) were taken out to study the phase morphology. At Position B, PP, PMVS and PMHS were mixed uniformly and the Pt catalyst was not added, which could be used to observe the morphology of the PP/SR-60 blend before dynamic-vulcanization. At Position C, SR was completely crosslinked. To prevent morphology changes, the samples were immediately quenched in liquid nitrogen after being taken out from HAAKE chamber. Figure 2B and C shows the TEM images of PP/SR-60 at the B and C position; the black part is SR, due to Si having a higher atomic number. The PP/SR-60 blend at Position B showed bicontinuous structure (Figure 2B) and the average phase size was about 0.5–1 μm. Figure 2C shows the sea–island structure of the PP/SR-60 blend after dynamic-vulcanization. The SR phase was the dispersed phase, and average size was 2.4 ± 1.2 μm, which demonstrated that phase inversion was completed in the dynamic-vulcanization process, and PP/SR-60 TPV was prepared. Moreover, it can be seen that the SR size observed by TEM was larger than that characterized by SEM. This may be attributed to the larger SR phase not being removed by the etching process; hence, SEM images did not show bigger holes (SR phase). 

Banerjee’s studies found that the rubber phase would decrease to nanosized rubber particles after injection (80 MPa), when they studied polyamide 6/fluoroelastomer TPVs [29,30]. Wu et al. also found that the rubber phase in TPV was the aggregates composed by rubber nanoparticles, when they studied EPDM/PP TPVs [4,28,31]. Therefore, hot-press molding was subjected to PP/SR-60 TPV to study the influence of the hot press on SR phase size. The mechanical sample that was prepared by hot press was sliced into thin films by cryo-ultramicrotome and used to observe the SR phase size by TEM. Figure 2D shows the morphology of PP/SR-60 TPV after the hot press. Comparing SR morphology in Figure 2C, the large-size SR (in red circles) disappeared, and SR size became smaller and more homogeneous, with the average SR size being 1.6 ± 0.7 μm. This may indicate that the SR phase in PP/SR TPV was rubber aggregates that were composed of smaller SR rubber particles, the hot press (10 MPa) could break the rubber aggregates, and make the large SR phase smaller. Furthermore, it should be noted that, because the hot-press molding process had a lower shear rate (1–10 s^-1^) than that of the injection-molding processes (1000 s^-1^) in Banerjee’s study, the rubber-phase size change was smaller in our study.

### 3.3. Strain Sweep of PP/SR TPVs

**Figure 4:**
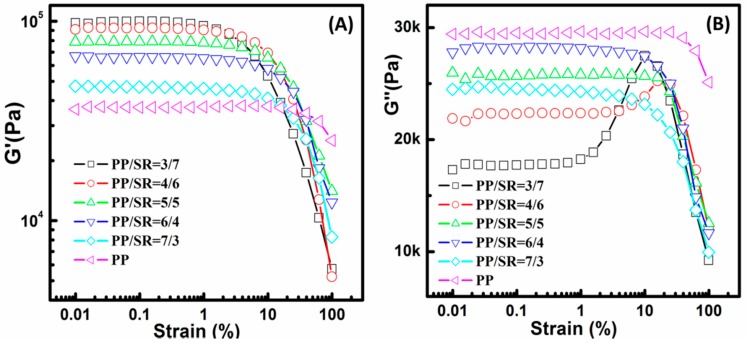
The dependence of the (**A**) dynamic storage modulus (*G*′) and (**B**) loss modulus (*G*″) on strain amplitude measured at 200 °C and 10 rad/s for different polypropylene (PP)/silicone rubber (SR) thermoplastic vulcanizates (TPVs).

Strain sweep was used to determine the linear viscoelastic region of TPVs and microstructure change at different strains [32,33]. Figure 4A and B show the dependence of the dynamic storage modulus (*G*′) and loss modulus (*G*″) on the strain amplitude measured at 200 °C and 10 rad/s for different PP/SR TPVs. Thermal history and degradation can be neglected for different PP/SR TPV samples because 200 °C is higher than PP’s melt point, and an antioxidant was added in every PP/SR TPV sample. Linear viscoelastic behavior corresponding to strain-independent *G*′ was observed at small-strain amplitudes for all samples. As shown in Figure 4A, a strain of 1%, used in frequency sweep, guaranteed the appearance of linear viscoelasticity under the tested conditions. It was found that the *G*′ increased as SR content increased due to SR being crosslinked and exhibiting elasticity in PP/SR TPVs. Moreover, the *G*′ decreasing rapidly with increasing strain was called the “Payne effect” in filler-reinforced rubber systems [33,34,35], and was attributed to the deformation of physical bonds linking adjacent filler clusters. In PP/SR TPVs, there were no covalent bonds between PP and SR, and the SR phase formed a physical SR network in the PP matrix due to the high ratio of SR and their interactions; therefore, the *G*′ decreasing with increasing strain should be attributed to the deformation of SR physical networks. 

On the other hand, it was noted that G″ decreased with the increase of SR content, and the *G*″ of PP/SR-60 and PP/SR-70 TPVs displayed a peak with strain in Figure 4B. The *G*″ viscously represented, and PP exhibited a viscous feature in PP/SR TPVs; therefore, the *G*″ decreased with SR content increasing (PP content decreased). The phenomenon of the *G*″ showing a peak in strain sweep tests was also well known as the “Payne effect” in rubber rheology studies, which was always attributed to the destruction and reconstruction of particle networks. According to the morphology study in the above section, SR rubbers were aggregates that were composed of smaller SR rubber particles and formed a physical network in the PP matrix; therefore, the *G*″ peak should be related to the destruction and reconstruction of SR rubber-particle aggregates. For high SR content, the SR network was easier to break into small SR aggregates, but also easier to rebuild due to a higher SR ratio (more contact). The breaking and rebuilding of the SR network needs energy, so the *G*″ would increase under proper strains. As the strain continued to increase, the rebuilding SR network became difficult, energy loss decreased, and the G″ began to decrease. Therefore, the *G*″ peak should correspond to the balance of destruction and reconstruction of SR rubber networks.

### 3.4. Viscoelastic Properties of PP/SR TPVs

**Figure 5:**
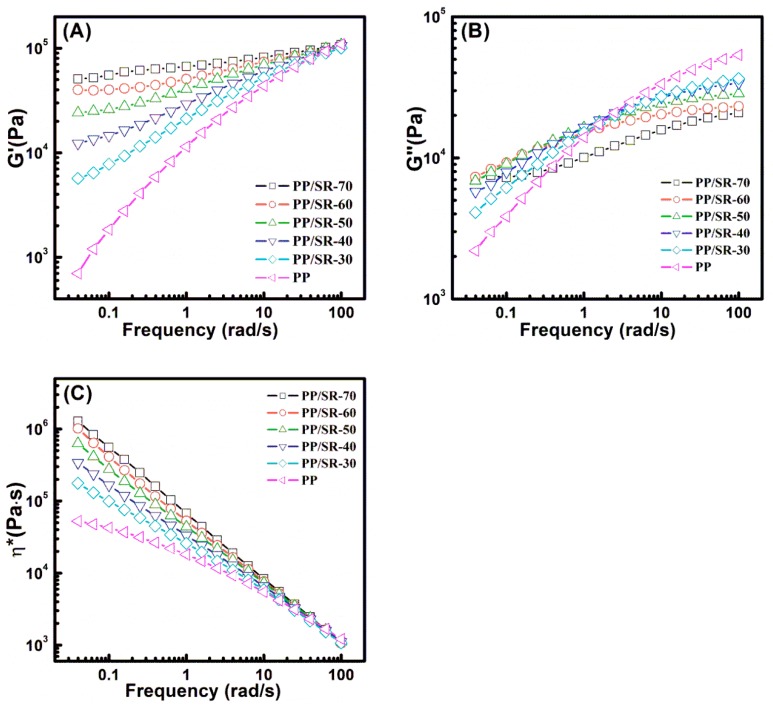
Plots of (**A**) *G*′, (**B**) *G*″ and (**C**) complex viscosity (η*) vs. frequency for polypropylene (PP) and PP/ silicone rubber (SR) thermoplastic vulcanizates (TPVs) with different SR content at 200 °C.

Figure 5A and B present the plots of *G*′ and *G*″ vs. the frequency for PP and PP/SR TPVs with different SR content under 1% strain at 200 °C. The *G*′ increased and the slope of *G*′ and ω at low frequencies decreased as SR content increased due to the elastic SR content increasing and the SR rubber network forming. The change of *G*″ was complicated. At a high frequency regime, the *G*″ decreased with SR content increasing; at a low-frequency regime, the *G*″ increased with SR content increasing. For PP/SR TPVs, the *G*″ is related to PP, which exhibits a viscous feature. Therefore, this phenomenon can be attributed to the movement limitation of PP chains by the SR phase. At high frequencies, the movements of PP chains were very small, and SR had little influence on PP chains movements. Hence, *G*″ increased with PP content increasing due to more PP chains moving and consuming energy. At low frequencies, the movements of PP chains were longer, and the limitation of PP chains by SR could not be neglected. The more SR content, the higher constraint on PP chain movements, and the more energy consumed. Hence, the *G*″ increased with SR content increasing.

Figure 5C shows the dependence of complex viscosity η* on frequency. According to the Cox–Merz rule, it can be used to reflect shear viscosity when the values of the shear rate and the oscillation frequency are equal. It can be seen that the η* of PP/SR TPVs increased and performed stronger shear thinning behavior with the increase of SR content. At a high-frequency regime, the η* of different PP/SR TPVs tended to show similar values, which suggests that high SR content did not affect PP/SR TPVs’ processability in extrusion molding, which had a high shear rate or shear stress. 

### 3.5. Creep Behaviors

**Figure 6:**
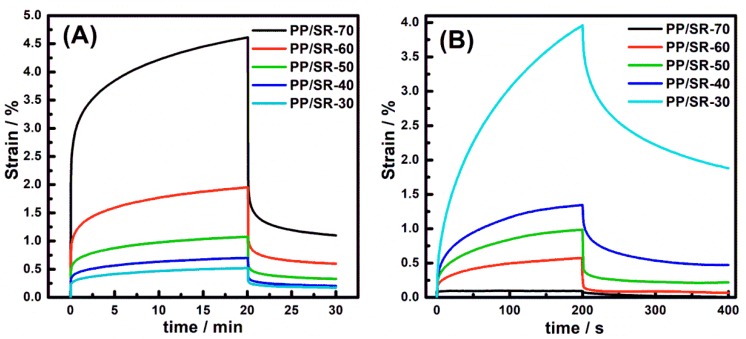
Creep deformation and its recovery behavior of polypropylene (PP)/silicone rubber (SR) thermoplastic vulcanizates (TPVs) at (**A**) 40 °C and (**B**) 180 °C.

Creep is the tendency of materials to deform under stress, which is quite important for the applications of materials requiring long-term durability and reliability. Figure 6A shows the PP/SR TPVs creep deformation and recovery behavior at 40 °C. It can be found that all PP/SR TPVs exhibited a primary creep with a high strain rate, and a secondary creep with a slow strain rate. Then, all PP/SR TPVs showed primary rapid creep recovery and secondary slow creep recovery. This was consistent with creep and its recovery plot for viscoelastic polymers. It also found that creep-deformation and creep-recovery values substantially increased with the increase of SR content, but the recovery rate was very similar for PP/SR TPVs, as shown in Table 2. 

On the other hand, Figure 6B shows PP/SR TPV melt creep deformation and recovery behavior at 180 °C, and it can be seen that the shear creep-deformation and creep-recovery values decreased with the increase of SR content. For PP/SR-30 TPV, creep deformation and recovery were different from other samples, which can be attributed to PP’s melt features. PP was melted and performed viscous features at 180 °C, and the PP/SR-30 sample had more PP content; therefore, it showed larger creep deformation at the same creep time and less recovery than others. Moreover, with SR content increasing, recovery rate increased, reaching 96%, which demonstrated that SR increased the elastic and dimensional stability of the PP/SR TPV melt. 

The different creep behavior of PP/SR TPVs at 40 and 180 °C can be explained as follows. For PP/SR TPVs at 40 °C, the SR had a lower modulus than that of PP; hence, creep deformation of PP/SR TPVs is determined by SR. The more SR content there is, the higher the creep deformation. However, the creep recoveries of PP/SR TPVs were dependent on the PP, because PP was difficult to recover, so all PP/SR TPVs showed similar recovery rates. For PP/SR TPVs melts at 180 °C, PP had a lower modulus. Therefore, creep deformation of PP/SR TPVs was determined by PP; the less the SR content, the higher the creep deformation. Moreover, SR was crosslinked and exhibited elasticity; hence, with the increase of SR content, the PP/SR TPV melt had a higher recovery rate.

## 4. Conclusions

PP/SR TPVs were prepared by dynamic-vulcanization technology. The mixing torque of PP/SR blends, viscoelastic properties, and creep response of PP/SR TPVs were studied. The size of the SR phase in TPVs was 2.4 μm after dynamic vulcanization by TEM observation, but the hot press would break the SR phase and make the large SR phase smaller, which means the SR phase was composed of smaller SR rubber particles and higher shear, being able to make the size of the rubber phase smaller and uniform. PP/SR TPVs showed a distinct “Payne effect”, which was attributed to the destruction and reconstruction of SR physical networks. Moreover, creep deformation and its recovery of PP/SR TPVs were dependent on different factors at 40 and 200 °C, respectively.

## Figures and Tables

**Table 1:** Formulations of the polypropylene (PP)/silicone rubber (SR) thermoplastic vulcanizates (weight ratio).

	Sample	PP/SR-30	PP/SR-40	PP/SR-50	PP/SR-60	PP/SR-70
Component						
PP		70	60	50	40	30
PMVS		30	40	50	60	70
Pt catalyst		0.06	0.08	0.10	0.12	0.14
PMHS		0.9	1.2	1.5	1.8	2.1
Alkynol inhibitor		0.3	0.4	0.5	0.6	0.7
Antioxidant		0.1	0.1	0.1	0.1	0.1

**Table 2:** Creep deformation, creep recovery and recovery rate values of polypropylene (PP)/silicone rubber (SR) thermoplastic vulcanizates (TPVs) at 40 and 180 °C.

Samples	40 °C			180 °C		
	Creep deformation (%)	Creep recovery (%)	Recovery rate (%)	Creep deformation (%)	Creep recovery (%)	Recovery rate (%)
PP/SR-30	0.52	0.17	67.3	3.96	1.88	52.4
PP/SR-40	0.70	0.20	71.1	1.34	0.47	64.9
PP/SR-50	1.08	0.33	68.7	0.98	0.26	73.4
PP/SR-60	1.95	0.60	69.8	0.57	0.07	88.4
PP/SR-70	4.61	1.13	75.4	0.10	0.0035	96.4
